# Invasion success of a scarab beetle within its native range: host range expansion versus host-shift

**DOI:** 10.7717/peerj.262

**Published:** 2014-02-25

**Authors:** Marie-Caroline Lefort, Stéphane Boyer, Saïana De Romans, Travis Glare, Karen Armstrong, Susan Worner

**Affiliations:** 1 Bio-Protection Research Centre, Lincoln University, Lincoln, Christchurch, New Zealand; 2 Université d’Angers, Angers, France

**Keywords:** Host-race, Biotype, Native invader, Scarab, Exotic host plant, *Costelytra zealandica*

## Abstract

Only recently has it been formally acknowledged that native species can occasionally reach the status of ‘pest’ or ‘invasive species’ within their own native range. The study of such species has potential to help unravel fundamental aspects of biological invasions. A good model for such a study is the New Zealand native scarab beetle, *Costelytra zealandica* (White), which even in the presence of its natural enemies has become invasive in exotic pastures throughout the country. Because *C. zealandica* still occurs widely within its native habitat, we hypothesised that this species has only undergone a host range expansion (ability to use equally both an ancestral and new host) onto exotic hosts rather than a host shift (loss of fitness on the ancestral host in comparison to the new host). Moreover, this host range expansion could be one of the main drivers of its invasion success. In this study, we investigated the fitness response of populations of *C. zealandica* from native and exotic flora, to several feeding treatments comprising its main exotic host plant as well as one of its ancestral hosts. Our results suggest that our initial hypothesis was incorrect and that *C. zealandica* populations occurring in exotic pastures have experienced a host-shift rather than simply a host-range expansion. This finding suggests that an exotic plant introduction can facilitate the evolution of a distinct native host-race, a phenomenon often used as evidence for speciation in phytophagous insects and which may have been instrumental to the invasion success of *C. zealandica*.

## Introduction

Plant introductions to novel habitats have occurred worldwide over hundreds of years to sustain human migrations and subsequent needs (Burnett *et al.*, 2012). Even today, the number of such introductions continues to increase, although attention has changed over recent decades from species that mainly sustain food production (Godfray *et al.*, 2010) to species that are introduced accidentally (McNeill *et al.*, 2011) or planted for amenity purposes (Brasier, 2008). As a result, a large variety of more or less complex relationships with the members of native communities have flourished (reviewed by Cox, 2004). Although these interactions often result in population declines among the native community (Ding & Blossey, 2009), sometimes the introduction of exotic plants can be taken as an opportunity by native species to expand and flourish outside of their native habitat. This can occur via the process of host range expansion (Mack *et al.*, 2000) and ultimately of host-shift, sometimes referred in the literature as host-switching (Agosta, 2006) or host-transference (Holder, 1990). Agosta (2006) defines a host-shift as the continuation of a host range expansion whereby a population of a phytophagous species forms an association with a novel host plant. In addition, Diegisser *et al.* (2009) specified that, in this process, the population which would have undergone the host-shift might not be able to use its new and its ancestral host simultaneously, which can be detected by a host-plant associated fitness trade-offs on the ancestral host (Via, 1990; Diegisser *et al.*, 2009). In contrast, host-range expansions do not result in such fitness compromises, allowing the population to use both its new and ancestral hosts (Diegisser *et al.*, 2009) without generating detrimental fitness response effect(s). We believe that these types of response are likely to be observed in native insects that sometimes reach the status of ‘pest’ or ‘invasive species’ on introduced plants.

In the last few years, Valéry *et al.* (2008*a*), Valéry *et al.* (2008*b*), Valéry *et al.* (2009), Valéry, Fritz & Lefeuvre (2013) debated the terminology relative to ‘biological invasion’ and demonstrated that it should not be solely confined to allochthonous species. For insects alone, and with more than 60 native species that have become notable for the economic damage that they cause (Scott, 1984), New Zealand is a perfect illustration of this assertion. In this country, the larval form of the native scarab *Costelytra zealandica* (White) (Coleoptera: Scarabaeidae) is certainly one of the most notorious local pests that attack numerous exotic plants (Given, 1966; East & Pottinger, 1984; Scott, 1984; Grimont *et al.*, 1988; Richards *et al.*, 1997), among which are several European-style pastoral plants such as clover and ryegrass. Despite this apparent luxuriant success on exotic hosts, this species still occurs widely within its native habitat, which is mainly composed of local fescue and tussock species. The present study aims to investigate whether the rise of *C. zealandica* as a native biological invader was driven simply by a host range expansion rather than by a complete host shift. The fitness response of two populations of *C. zealandica* was investigated through survivorship and weight increase of third instar larvae the longest and final larval stage in this species, under several feeding treatments comprising an exotic host plant as well as one of its ancestral hosts.

## Material and methods

### Insect sampling and plant culture

Two collection sites were selected, both in the South Island of New Zealand. In February 2012, young third instar larvae of the univoltine scarab *C. zealandica* were sampled at Hororata (43°32′17′′S 171°57′16′′E) and Cass (43°02′10′′S 171°45′40′′E), labeled as sites A and B respectively. Site A comprised typical European-style pastoral plant species dominated by exotic ryegrass and clover. In contrast, site B was essentially composed of New Zealand native tussock and fescue plant species.

All collected larvae were initially placed individually in ice tray compartments with a small piece of carrot as food and maintained at 15°C for four days to test for the presence of amber disease, the most common disease in this species (Jackson, Huger & Glare, 1993). Subsequently, healthy larvae were identified to the species level based on Hoy & Given’s 1952 description of the genus and on the morphology of their raster (Lefort *et al.*, 2013). For a few specimens for which morphological identification was difficult, a rapid diagnostic confirmation was made using a non-invasive molecular sampling method based on the use of frass as a source of DNA (Lefort *et al.*, 2012). All larvae were then randomly assigned to the various experimental treatments.

The introduced white clover (*Trifolium repens*) was used as an exotic host to rear and feed the larvae of *C. zealandica*. It was grown from seeds (PGG Wrightson Seeds Ltd, Christchurch, NZ) in a glasshouse in 200 ml of potting mix comprising 60% peat and 40% sterilized pumice stones. Young plants of the native *Poa cita* (silver tussock) were purchased from Trees for Canterbury (Christchurch, NZ) and used as ancestral native host. Each plant was carefully transferred from its original pot to a 200 ml pot, filled with potting mix comprising 60% peat and 40% sterilized pumice stones, and was allowed to grow for two months in a glasshouse.

### Native versus exotic hosts and artificial host-shift experiment

Following identification, *C. zealandica* larvae (*n* = 180) were weighted and placed in individual 35 ml plastic containers containing 50 g of gamma-irradiated soil (Schering-Plough Animal Health, Wellington, NZ). Containers were randomly allocated to three trays so as to create 10 blocks, where the larvae were ordered from the lowest to the highest weight on the trays to allow the detection of confounding factors effects. Each container was randomly assigned to a feeding treatment. Feed trials were performed at 15°C over a period of 12 weeks corresponding to the most intense feeding period of the third instar larval stage in *C. zealandica*. Larvae were fed *ad libitum* with freshly chopped roots of the selected host plant. They were either fed with clover or tussock for 12 weeks respectively for treatments 1 (T1) and 2 (T2), or with tussock for 7 weeks followed by a shift of 5 weeks on clover for treatment 3 (T3).

The fitness response of the larvae was evaluated by measuring survivorship and percentage increase in weight on a weekly basis. Statistical tests were conducted with R software (R Development Core Team, 2009) and GenStat® (GenStat 14, VSN International Ltd, UK).

Statistical analyses on the effect of each host plants (T1 and T2) and of the artificial host shift (T3) on larval survival were carried out using a Chi-squared test. The treatment effect (T1, T2 and T3) on larval growth was analyzed by analysis of covariance (ANCOVA), with the larvae initial weight used as a covariate. The latter analysis was performed after exclusion of larvae that died before the end of the 14 weeks of data collection.

## Results

### Larval survival

**Figure 1:**
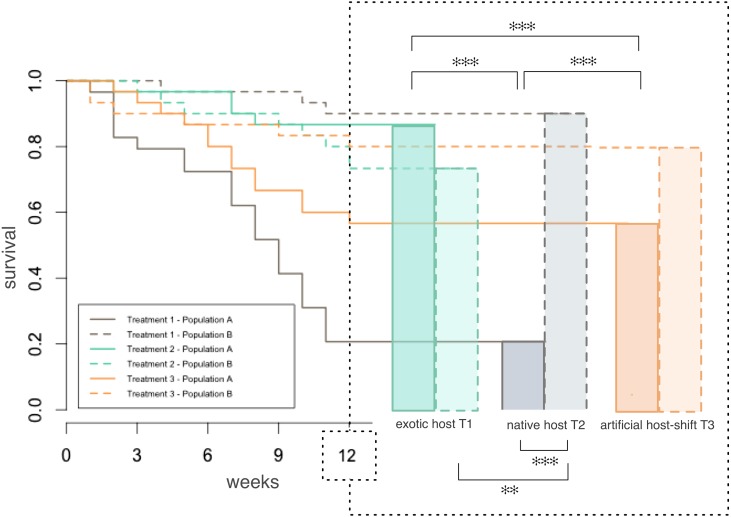
Larval survival of two populations of *Costelytra zealandica* during 12 weeks of feeding treatment with tussock, clover or with a combination of the two plants. Kaplan Meier plot of survival during the 12 weeks of feeding treatment. Right: final survival after 12 weeks. Population A (dark colored bars) was collected from exotic pastures and population B (light colored bars) was collected from New Zealand native grasslands. All pairwise comparisons were performed using chi-squared tests after 12 weeks of treatment. Only significant differences are indicated on the figure (*p* < 0.001∗∗∗ and *p* < 0.01∗∗).

Death events occurred regularly over the 12 weeks of treatment in each treatment and for both populations studied (Fig. 1). After 12 weeks, the larvae collected from exotic pastures (population A) displayed significantly better survival rates when fed with the exotic host plant (T2, 86% survival) as opposed to their native host (T1, 20% survival) (*χ*^2^ = 86.6364, d.f. = 1, *p* < 0.001) (Fig. 1). Similarly, these larvae survived significantly better when fed with a combination of native followed by exotic host plants (T3, 56% survival) than when fed with their native host only (T1) (*χ*^2^ = 26.9118, d.f. = 1, *p* < 0.001).

In contrast, no significant survival differences were detected for the larvae collected from native grasslands (population B) across all treatments (Fig. 1) (Chi-squared tests respectively T1/T3 *χ*^2^ = 3.1765, d.f. = 1, *p* = 0.074 71, and T2/T3 *χ*^2^ = 0.8985, d.f. = 1, *p* = 0.3432).

### Larval growth

**Figure 2:**
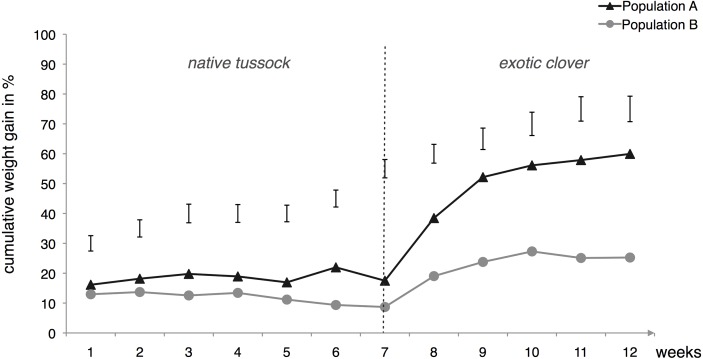
Cumulative weight gain of two populations of *Costelytra zealandica* larvae following 12 weeks of artificial host-shift feeding treatment, where larvae were fed for 7 weeks on tussock and 5 weeks on clover. Population A (dark grey line) (*n* = 17) was collected from exotic pastures and population B (light grey line) (*n* = 24) from New Zealand native grasslands. Vertical bars represent 5% LSDs (Least Significant Difference) at the end of each week of treatment.

When the larvae were exposed to the artificial host-shift feeding treatment (T3), and fed with native tussock during the first phase of the experiment, no differences in terms of weight gain were detectable between the two populations studied (Fig. 2). However, this trend changed considerably after the host-shift that occurred in week 7. Larvae belonging to the population collected from exotic pastures (population A) quickly increased weight by over 40% during the second phase of treatment that lasted for 5 weeks, which was significantly more than population B larvae that only increased their weight by about 16.5% (Fig. 2).

**Figure 3:**
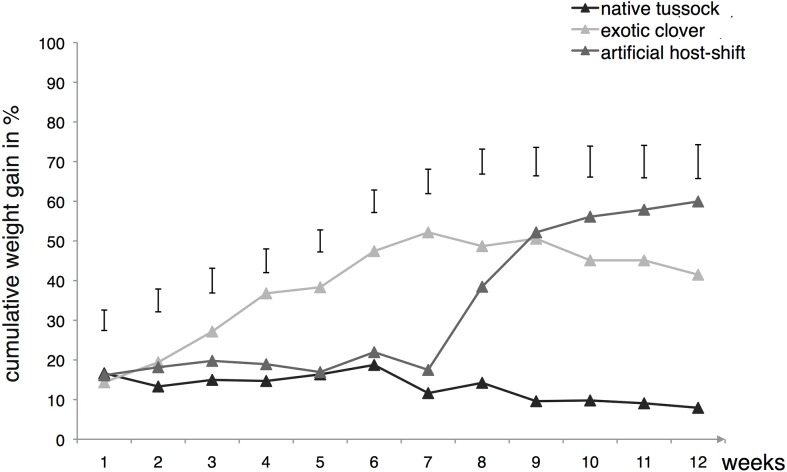
Cumulative weight gain of *Costelytra zealandica* larvae collected from exotic pasture following 12 weeks of feeding treatment on various host plants. The native tussock feeding treatment (T1) appears in dark grey (*n* = 6), the clover feeding treatment (T2) in light grey (*n* = 26) and the artificial host-shift feeding treatment (T3) in medium grey (*n* = 17). Vertical bars represent 5% LSDs (Least Significant Difference) at the end of each week of treatment.

It appeared that population A responded much better to the exotic host feeding as shown by the rapid increase in weight just after the host-shift in T3, and also by an overall weight gain close to 60% for the larvae submitted to T1 (Fig. 3). In contrast, when population A was kept feeding on native tussock for 12 weeks (T2), larvae lost a significant amount of weight (Fig. 3). From week 8 onward, the differences resulting between this treatment (T2) and the exotic based treatments (T1 and T3) were highly significant (all weeks, ANCOVA, *p* values < 0.001) (Fig. 3).

## Discussion

An important challenge for ecologists and evolutionary biologists is to investigate the various contributing factors to biological invasions. Among these are the processes by which some species reach the status of invaders in their home range. The present study aimed to address the identification and investigation of such drivers in *C. zealandica*. Our results recorded the existence of strong intra-specific variations in fitness of this species. These variations were expressed as important differences in survivorship and weight increase when different larval populations, recovered from different host plants and regions, were exposed to their ancestral native or exotic host plants.

An overall high fitness performance was observed on clover, expressed as high survivorship and high larval weight increase, by *C. zealandica* collected from exotic pastures. As discussed elsewhere, such results may reflect some sort of inheritance and maternal effect (Mousseau & Dingle, 1991; Mousseau & Fox, 1998), where the offspring of a given population is expected to display high fitness performances (Fox, 2006) and similar host preferences as their parents (Craig, Horner & Itami, 2001). However, for this particular species, neither inheritance traits or maternal effect, nor an alternative explanation such as the high nutritional value of clover (Awmack & Leather, 2002), can explain the observed increased performances of the larvae (Lefort, 2013). Nevertheless, it is quite likely that intrinsic mechanisms relying on high degrees of phenotypic plasticity, such as variation in host tolerances (Agrawal, 2000; Kant *et al.*, 2008) rapid adaptation (i.e., evolutionary host-shift) (Holder, 1990; Menken & Roessingh, 1998; Agosta, 2006) or ecological fitting sensus Agosta (2006) (i.e., ecological host-shift), might be partially or totally responsible for the high fitness performance observed in *C. zealandica* collected from exotic pastures and fed on clover. Agosta (2006) defined the term ecological host-shift as a process that occurs through that of a host range expansion, whereby an organism is able to use new resources at the moment of contact because of a latent ability that results in a novel association of species, and where consequently evolution by either member of the association shall not be a prerequisite. Because all the larvae of *C. zealandica*, regardless of their origin, displayed high survival rates when fed with clover as a ‘*new*’ host, this latter explanation appears appropriate. Furthermore, Holder (1990) suggested that this type of association often arises because of the physical proximity of the ancestral and the new host-plant species, a scenario that followed the European settlement in New Zealand, when numerous native forests and grasslands were replaced by exotic pastures and crops (McDowall, 1994; Lee, Allen & Tompkins, 2006). Effectively, this pattern of early settlement modification of the New Zealand landscape resulted in new ecological configurations where native grasslands ended up neighboring exotic cultures and grass pastures. It is believed that this physical proximity has resulted in the contraction of native plant distribution ranges and in the exploitation of these new modified habitats by native species (Yeates, 1991), as possibly observed in *C. zealandica* as an ecological host-shift.

Another tangible explanation for the exploitation of both native and newly exotic host plants by *C. zealandica* could be that this species has not yet undergone a host shift but only a host-range expansion onto exotic pastoral plants. This explanation is likely because of the close relationship that exists between this process and that of an ecological host-shift, and where, in both cases, no significant adaptation to the newly encountered exotic host is required (Diegisser *et al.*, 2009; Agosta, Janz & Brooks, 2010). However, the differences in fitness performances between the two populations of *C. zealandica*, which were observed following the ancestral host feeding treatment, refute this possibility and suggest another explanation. The larvae originating from exotic pastures seem no longer able to properly benefit from their ancestral host, as shown by very high mortality rates and low weight increase of the surviving larvae of this population. This fitness compromise, which is expressed as a host-plant associated fitness trade-off (Via, 1990; Diegisser *et al.*, 2009) resulting in some degree of maladaptation to the ancestral host plant of this species, is not compatible with the solely host range expansion theory and reinforces that of a host-shift occurrence (Diegisser *et al.*, 2009) for the population originating from exotic pastures.

Even though the ecological host-shift theory appears to conform to this case study, the slight variation in terms of weight gain between the two populations, following the artificial host-shift on clover suggests that some level of evolutionary change has occurred for the population collected from exotic pastures. Heard & Kitts (2012) suggested that host-shifts can be followed by host-associated differentiations that can result in the evolution of new biotypes of specialist races, or so-called host-races (Diehl & Bush, 1984; Drès & Mallet, 2002). Over the last decades, numerous examples of host-race formation in insects have been described. Amongst the most recent examples, Downey & Nice (2011) reported the possibility of ongoing host-race formation in the juniper hairstreak butterfly (*Callophrys gryneus*), following the observation of differential larval fitness performances when reared on natal versus alternate hosts. More recently, Bourguet *et al.* (2014) suggested ecological speciation as a possible evolutionary scenario leading to reproductive isolation between the Asian and the European corn borers in the genus *Ostrinia* (Lepidoptera, Crambidae). Using molecular tools, they concluded that the process by which these borers became agricultural pests could have lead to the emergence of these two distinct species from one ancestral species. The results of the present study strongly suggest a similar scenario, where an ecological host-shift in at least one population of *C. zealandica* would have led to the emergence of distinct host-races in this species. Hence, it is likely that the invasive *C. zealandica* might solely represent a particular biotype. Any phenotypic plasticity that initially facilitated the assumed host-shift and host-race formation, could, in the long term, lead to speciation (e.g., West-Eberhard, 1989; Agrawal, 2000; Agosta, 2006; Heard & Kitts, 2012) in this insect. Furthermore, these findings point to a very interesting case of sympatric host races formation facilitated by exotic plant introductions, and resulting in the rise of a phytophagous insect to the rank of invasive species in its own native range.

To summarise, this study has shown evidences of (1) host-shift initiation by host range expansion in *C. zealandica*, upon contact with exotic host plant, given the ability of the populations of *C. zealandica* recovered from native grasslands to perform well on exotic host plant, followed by (2) host-shift completion in the population collected from exotic pastures, where some level of evolutionary change have prevailed in populations feeding on an exotic host plants, until the ability to effectively use the native host has been lost and have resulted in (3) the formation of distinct host-races in *C. zealandica*.

## Additional Information and Declarations

### Field Study Permissions

The following information was supplied relating to ethical approvals (i.e., approving body and any reference numbers): Richard Townsend and Canterbury University granted access to the different insect collection sites.
